# Over-the-scope clip to the rescue: solution for duodenal perforation from migrated biliary stent

**DOI:** 10.1016/j.vgie.2022.11.007

**Published:** 2023-02-09

**Authors:** Chloe Tom, Wissam Kiwan, Omar Bakr, Jennifer Phan

**Affiliations:** Keck School of Medicine of USC Division of Gastrointestinal and Liver Diseases, Los Angeles, California

**Keywords:** OTSC, over-the-scope clip

## Abstract

Video 1 Over-the-scope clip to the rescue: solution for duodenal perforation from migrated biliary stent.

Over-the-scope clip to the rescue: solution for duodenal perforation from migrated biliary stent.

## Introduction

Iatrogenic perforation of the duodenum caused by biliary stents is a rare adverse event of ERCP.^1^ While the only available definitive treatment option was previously surgery, the advancement in the minimally invasive endoscopic field led to the development of over-the-scope clips (OTSCs), which allow complete closure of full-thickness defects endoscopically.^2^

## Case 1

A 66-year-old man was referred to our institution to resume management of choledocholithiasis and indeterminate biliary stricture. Eighteen months prior, he had jaundice secondary to bile duct stones and mid-distal common bile duct stricture for which he underwent ERCP and plastic stent placement. The COVID-19 pandemic led to delay in the necessary follow-up.

**Figure 1:**
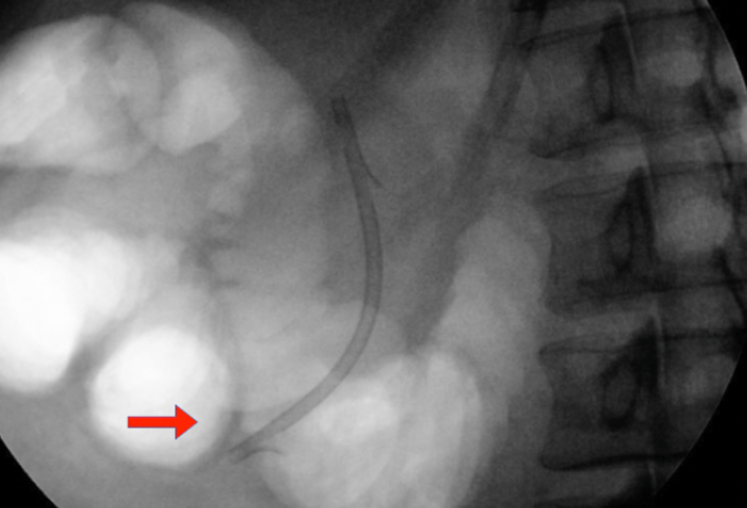
The fluoroscopic image confirmed the perforation, and approximately 1 cm of the stent extended outside the duodenal lumen and defect measured 4 mm in diameter.

**Figure 2:**
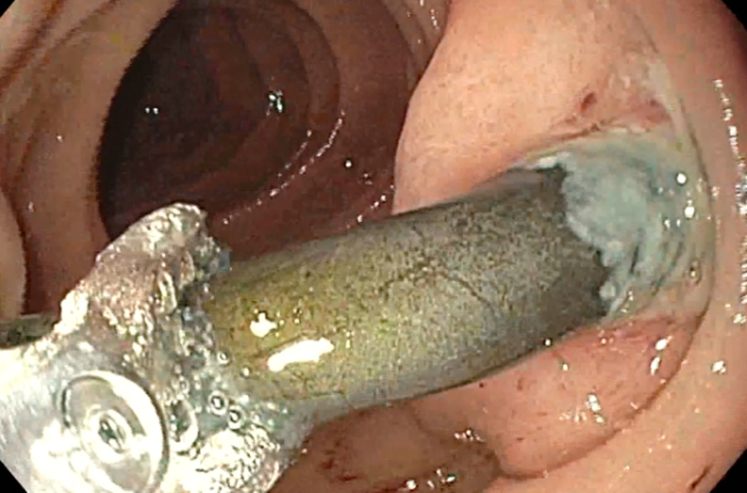
The stent was carefully extracted with a rat-tooth grasper.

**Figure 3:**
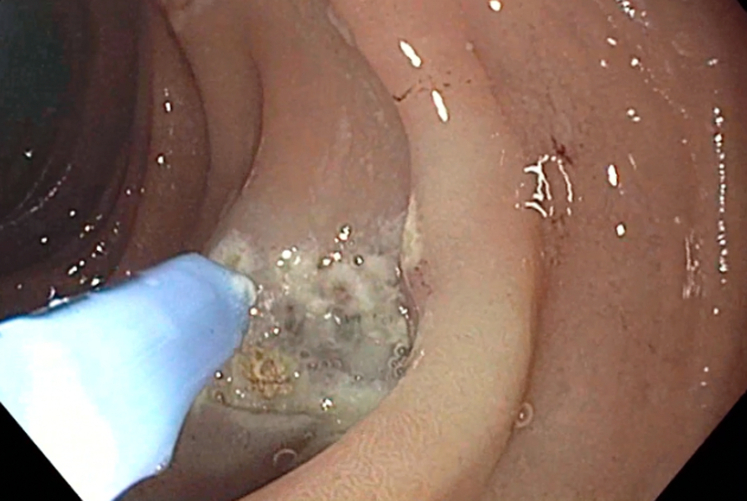
Coagulation for de-epithelialization of the surrounding tissue using argon plasma coagulation was successful.

**Figure 4:**
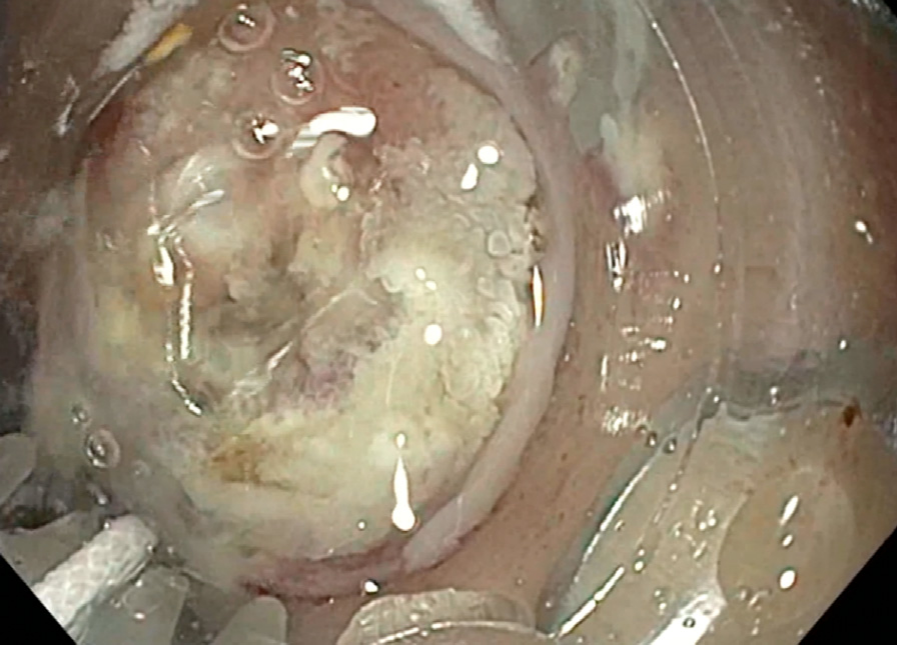
Gastroscope with distal clip attachment was reinserted and the tissue was brought into the cap via dynamic suctioning.

**Figure 5:**
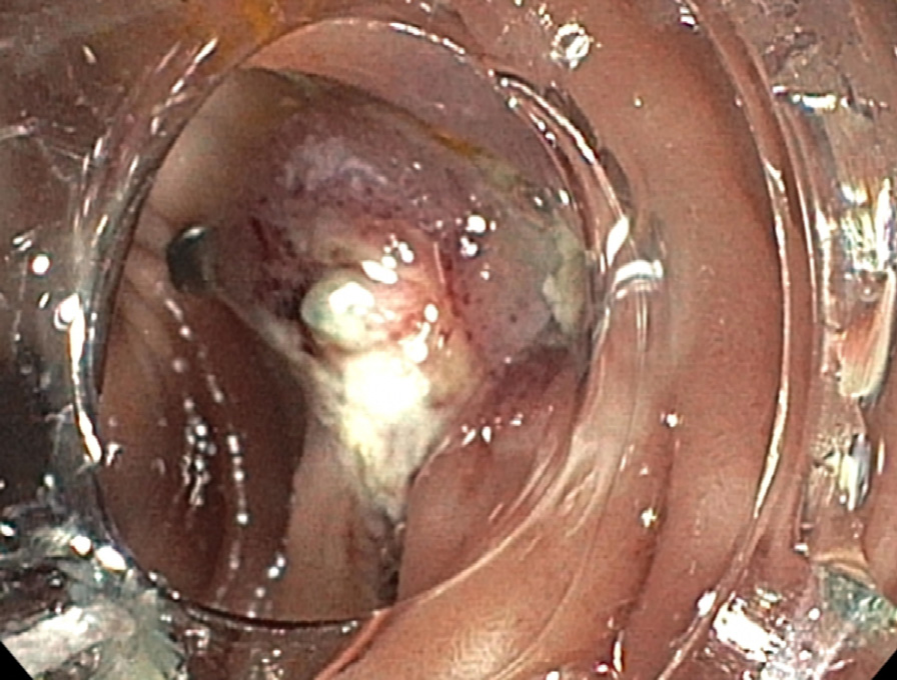
With the tissue in the cap, successful clip deployment and defect closure were performed.

**Figure 6:**
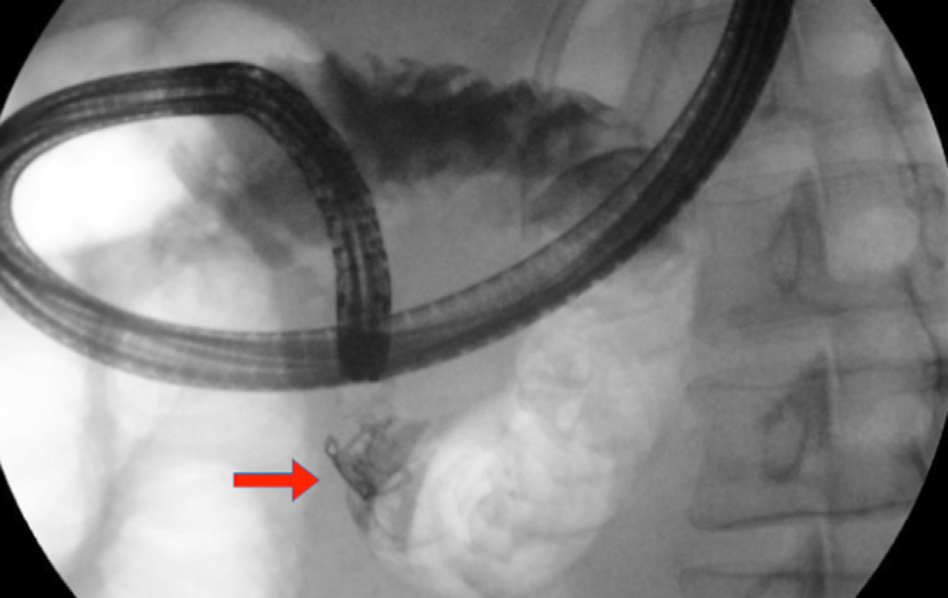
Postprocedural fluoroscopic image showed lack of contrast extravasation and confirmed complete closure.

Examination during EGD and upper EUS showed an incidental finding of a biliary stent emerging from the major papilla and penetrating the opposite duodenal wall (type 1 perforation) (Video 1, available online at www.giejournal.org). The fluoroscopic image confirmed the perforation and approximately 1 cm of the stent extended outside the duodenal lumen. The defect measured 4 mm in diameter (Fig. 1; Video 1). The stent was pulled with a rat-tooth grasper under fluoroscopy (Fig. 2; Video 1). Coagulation for de-epithelialization of the surrounding tissue using argon plasma coagulation (Erbe, Marietta, Ga, USA) was successful (Fig. 3; Video 1). Although this step is not required, it can assist with de-epithelization and help with locating the defect after stent removal. An OTSC (OTSC 11/6T Clip; OVESCO, Tuebingen, Germany) was used. The gastroscope (GIF-H190; Olympus, Center Valley, Pa, USA) was readvanced and the defect was visualized. The tissue was carefully suctioned into the cap to allow successful clip deployment and defect closure (Figs. 4 and 5; Video 1). A lack of contrast extravasation confirmed complete closure (Fig. 6; Video 1).

**Figure 7:**
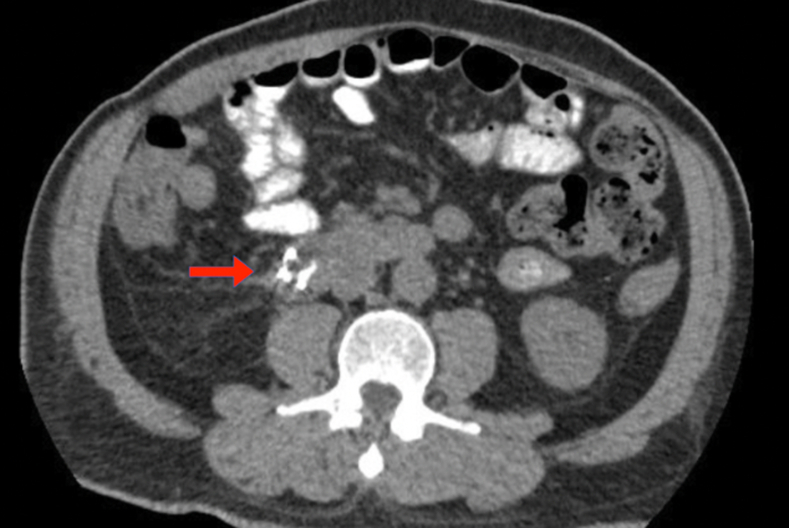
A postprocedural CT scan showed the clip in the proper place and no extraluminal fluid collection, air, or contrast extravasation.

A postprocedural CT scan showed no evidence of drainable fluid collection or extraluminal gas (Fig. 7; Video 1). The patient had no immediate or delayed adverse events. At his 2-month follow-up, he denied any pain, nausea, emesis, weight loss, or jaundice.

## Case 2

**Figure 8:**
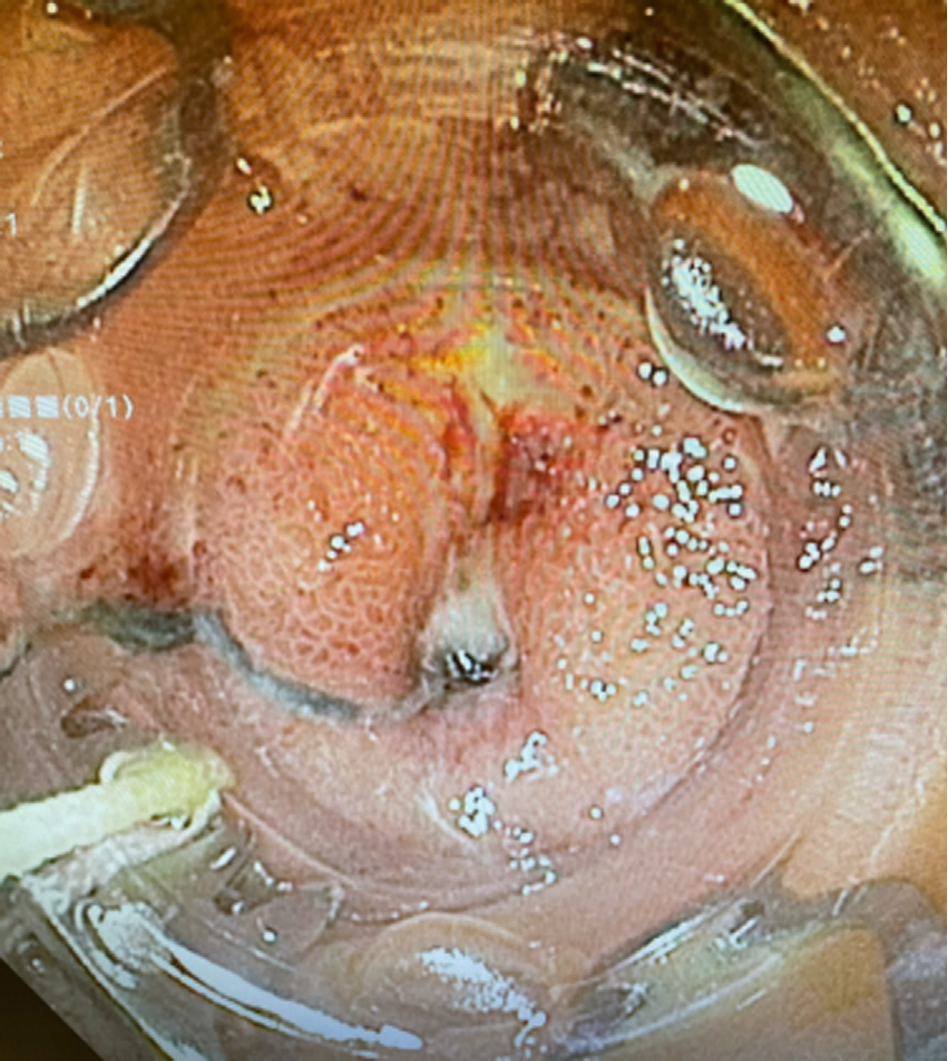
Over-the-scope-clip closure of duodenal defect.

A 71-year-old woman presented to an outside hospital with painless jaundice requiring ERCP and bilateral plastic biliary stent placement. Five days later, she was referred to our hospital for duodenal perforation from a migrated biliary stent with retroperitoneal fluid collection seen on a CT scan. She underwent a similar approach with removal of the stent via rat-tooth forceps and primary endoscopic closure of the duodenal defect with tissue ablation with argon plasma coagulation and OTSC closure (Fig. 8; Video 1). She received antibiotics and percutaneous drain placement into the fluid collection. Again, the closure remained intact.

## Case 3

**Figure 9:**
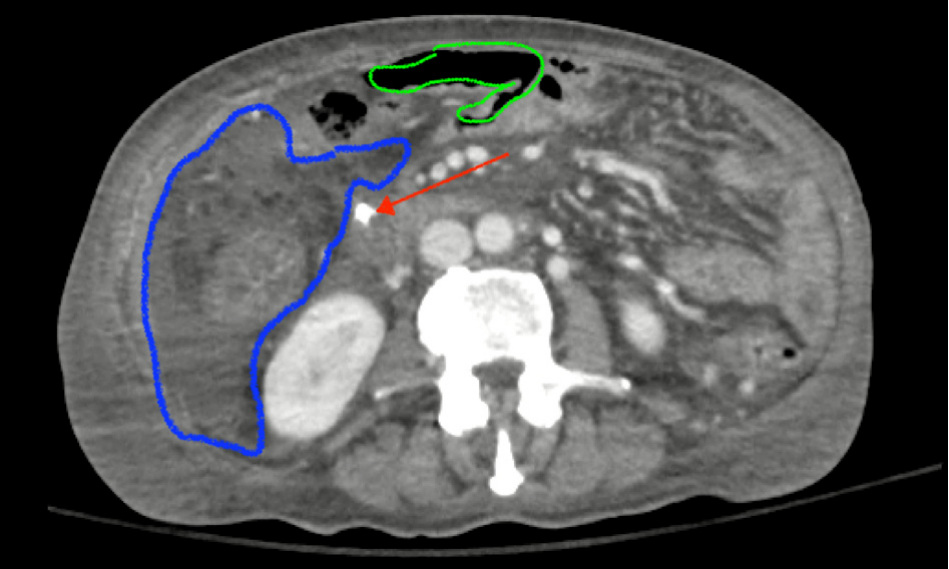
Perforation of a common bile duct stent (*red arrow*) through the second/third portion of the duodenum with intra-abdominal free fluid (*blue outline*) and air (*green outline*).

A 71-year-old woman with metastatic pancreatic adenocarcinoma presented with septic shock and encephalopathy, and she was found to have a migrated biliary stent with duodenal perforation. Her course was complicated by multiple intra-abdominal abscesses, extensive pneumoperitoneum (Fig. 9; Video 1), and was deemed not a surgical candidate given disease severity.

She underwent an ERCP that showed a migrated biliary stent causing opposing duodenal wall perforation. The defect site was ablated and an OTSC mended the perforation. Antibiotics were continued and she underwent percutaneous drain placement with radiology. There were no immediate adverse events and she had improvement in pain and mentation.

## Conclusion

Duodenal perforations from migrating plastic biliary stents are rare; however, they can be significantly morbid when they occur. While the presentations and time intervals between original stent placement to identification of duodenal perforation differed among the 3 cases (Table 1; Video 1), we found that endoscopic closure with OTSCs is an excellent option for intestinal perforation as a definitive treatment or bridge to further management.

**Table 1:** Case comparison

	Case 1	Case 2	Case 3
**Age, sex**	66, male	71, female	71, female
Clinical presentation	No significant history, presented with mild generalized abdominal pain	History of cholangiocarcinoma with biliary obstruction status after drainage with stents and colon cancer status post–partial colectomy, presented with obstructive painless jaundice with nausea, anorexia, and weight loss	History of metastatic pancreatic adenocarcinoma on palliative chemotherapy, presented with septic shock and encephalopathy
Abnormal cross-sectional imaging on presentation	Incidental finding of a biliary stent emerging from the major papilla and penetrating the opposite duodenal wall (type 1 perforation)	Duodenal perforation from a migrated biliary stent with concomitant retroperitoneal fluid collection seen on a CT scan	Migrated biliary stent with duodenal perforation and multiple intra-abdominal abscesses, and extensive pneumoperitoneum
Indication for biliary stent	Obstructive jaundice, choledocholithiasis, and indeterminate bile duct stricture	Malignant extrahepatic bile duct obstruction	Malignant extrahepatic bile duct obstruction
Interval time between biliary stent placement and diagnosis of perforation	18 months	5 days	10 weeks
Closure technique	Argon plasma coagulation used to demarcate perforation, rat-tooth grasper to remove stent, OVESCO 11/6T over-the-scope clip		
Clinical outcome	Successful closure without drainable fluid collection or extraluminal gas after the procedure. At 2-month follow-up he denied any pain, nausea, emesis, weight loss, or jaundice.	Successful closure, resolving jaundice, and down-trending bilirubin. A few days later she underwent uncomplicated surgical resection of the tumor along with partial hepatectomy.	Successful closure and remained on low-dose norepinephrine, stress-dose steroids, and meropenem and was deemed stable for transfer. She was not in our network and was not seen in a follow-up.

## Disclosure

*Dr Phan is a consultant for Boston Scientific, Olympus, and Noah Medical. All other authors disclosed no financial relationships.*

## Supplementary data

Video 1 Over-the-scope clip to the rescue: solution for duodenal perforation from migrated biliary stent.
